# A Stabilizer Framework for the Contextual Subspace Variational Quantum Eigensolver and the Noncontextual Projection Ansatz

**DOI:** 10.1021/acs.jctc.2c00910

**Published:** 2023-01-23

**Authors:** Tim Weaving, Alexis Ralli, William M. Kirby, Andrew Tranter, Peter J. Love, Peter V. Coveney

**Affiliations:** † Centre for Computational Science, Department of Chemistry, University College London, LondonWC1H 0AJ, United Kingdom; ‡ Department of Physics and Astronomy, Tufts University, Medford, Massachusetts02155, United States; ¶ Cambridge Quantum Computing, 9a Bridge Street, CambridgeCB2 1UB, United Kingdom; § Computational Science Initiative, Brookhaven National Laboratory, Upton, New York11973, United States; ∥ UCL Centre for Advanced Research Computing, Gower Street, LondonWC1E 6BT, United Kingdom; ⊥ Informatics Institute, University of Amsterdam, Amsterdam1098 XH, the Netherlands

## Abstract

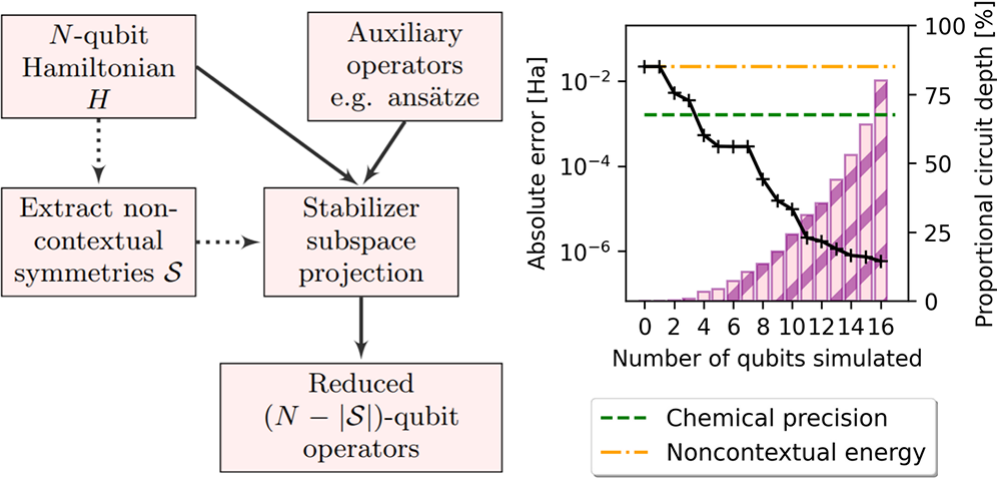

Quantum chemistry is a promising application for noisy intermediate-scale quantum (NISQ) devices. However, quantum computers have thus far not succeeded in providing solutions to problems of real scientific significance, with algorithmic advances being necessary to fully utilize even the modest NISQ machines available today. We discuss a method of ground state energy estimation predicated on a partitioning of the molecular Hamiltonian into two parts: one that is *noncontextual* and can be solved classically, supplemented by a *contextual* component that yields quantum corrections obtained via a Variational Quantum Eigensolver (VQE) routine. This approach has been termed *Contextual Subspace VQE* (CS-VQE); however, there are obstacles to overcome before it can be deployed on NISQ devices. The problem we address here is that of the ansatz, a parametrized quantum state over which we optimize during VQE; it is not initially clear how a splitting of the Hamiltonian should be reflected in the CS-VQE ansätze. We propose a “noncontextual projection” approach that is illuminated by a reformulation of CS-VQE in the stabilizer formalism. This defines an ansatz restriction from the full electronic structure problem to the contextual subspace and facilitates an implementation of CS-VQE that may be deployed on NISQ devices. We validate the noncontextual projection ansatz using a quantum simulator and demonstrate chemically precise ground state energy calculations for a suite of small molecules at a significant reduction in the required qubit count and circuit depth.

## 1 Introduction

Quantum computers promise to yield solutions to complex problems that have previously been unattainable by classical means, yet experimental demonstration remains challenging. To date, some of the largest molecules simulated on noisy intermediate-scale quantum (NISQ) hardware are H_12_–albeit only a Hartree–Fock calculation–conducted by Google using just 12 of the 53 qubits available on their superconducting quantum processor *Sycamore*,^1^ and H_2_O performed independently by IonQ using 3 qubits of an unspecified proprietary trapped ion device^2^ and by IBM using 5 of the 27 qubits on the now-decommissioned *ibmq_dublin* superconducting device.^3^

Due to the limitations of short coherence times, restrictive qubit connectivity and high noise floors that characterize the NISQ era, we are not able to harness the full state-space afforded to these machines. To circumvent the above issues, we turn to the class of variational quantum algorithms, of which the Variational Quantum Eigensolver (VQE)^4^ is most widely studied. In contrast with eigenvalue-finding algorithms requiring fault-tolerant machines such as Quantum Phase Estimation (QPE),^5^ which necessitates state evolution over an extended period of coherence, VQE executes a large ensemble of comparatively shallow parametrized circuits to estimate energy expectation values, informing a classical optimizer that updates the parameter settings before reinitialization of the quantum circuit. Its success is predicated on the variational principle, meaning the ground state energy of the system bounds expectation values from below.^6^

However, VQE is not without its challenges. First of all, the parametrized quantum state mentioned above, known as an *ansatz*, needs to be constructed carefully: It must be sufficiently expressible so the subspace of quantum states it spans contains the true ground state. On the other hand, if the ansatz is too expressible, we run into the problem of barren plateaus^7^ where we observe vanishing gradients. This is more often a symptom of “hardware efficient” ansätze,^1,8−12^ which aim to access the largest possible region of Hilbert space for the fewest number of native quantum gates.

To avoid barren plateaus, one must take into account some of the underlying problem structure to define ansatz circuits whose images are confined to a smaller but more targeted region of Hilbert space. Within this category are “chemically inspired” ansätze that represent sequences of electronic excitation operators in circuit; unitary coupled cluster (UCC)^13,14^ is widely acknowledged as the gold standard for electronic structure simulations, albeit computationally very expensive in practice.

More recently, we have seen the development of hybrid ansätze that bridge the gap between hardware efficiency and chemical motivation. For example, Gard et al.^15^ designed a compact circuit designed to conserve molecule symmetries such as particle number and spin, while Adaptive Derivative-Assembled Pseudo-Trotter (ADAPT) VQE^16−19^ describes a more complete approach to scalable quantum chemistry simulations by defining selection criteria of ansatz terms from a pool of excitation operators.

Second, the energy estimation procedure in VQE invokes the measurement problem; in order to mitigate statistical error, many prepare-and-measure cycles are necessary to achieve sufficient precision in the estimate. The advances made in recent years toward measurement reduction techniques are expansive^20−29^ and range from classical pre/postprocessing of the measurement information, such as in classical shadow tomography,^30,31^ to Hamiltonian term-grouping schemes and reductions in the number of Hamiltonian terms at a cost of coherent resource, such as in unitary partitioning.^32−36^ Combined with techniques of error mitigation,^37−44^ one can optimize VQE with the objective of maximal NISQ resource utilization.

In this work, we are concerned with Contextual Subspace VQE (CS-VQE),^45^ which describes a method of partitioning the molecular Hamiltonian into disjoint parts so that an electronic structure problem may be simulated to some degree on the available quantum device, even when the dimension of the full problem is too great to be encoded on the number of qubits available. This is supplemented by some classical overhead, but this often permits one to achieve chemical precision (to within 1.6 mHa ≈ 4 kJ/mol of the full configuration interaction (FCI) energy) at a saving of qubits, as indicated by Kirby et al.^45^ We highlight the relevance of chemical *precision* over *accuracy* here; since a minimal basis set (STO-3G) will be used throughout our benchmark, one should not expect agreement with experimentally obtained molecular energies and thus chemical accuracy is not an appropriate phrase. A finer basis set such as cc-pV*x*Z where *x* = D, T, Q, etc. should be used if one wishes to assess accuracy; however, this comes at the cost of increased qubits.

There has since been further research into the use of classical estimates of the electronic structure problem to reduce the resource requirements on quantum hardware. In particular, Classically Boosted VQE (CB-VQE)^46^ identifies classically tractable states and excludes them from the quantum simulation, alleviating some measurement and fidelity requirements of the VQE routine. CS-VQE also bears a resemblance to the qubit reduction technique of qubit tapering,^47,48^ which exploits  symmetries of the Hamiltonian; the differences and similarities are highlighted herein and by Kirby et al.^45^

There are still a number of problems to address before CS-VQE may be successfully deployed on real quantum hardware, most notably with regard to the ansatz, which is the principal focus of this work. To aid this objective, we place the method on a strong theoretical footing of stabilizer subspaces and projections therein; this reformulation is better suited to efficient implementation, which is being addressed through the *Symmer* project.^49^ This rephrasing of CS-VQE illuminates the matter of constructing ansätze for the contextual subspace and renders this method compatible with contemporary approaches to ansatz construction such as ADAPT-VQE.

## 2 Preliminaries

The notation used throughout shall be to write operators in standard capital font (*A*, *B*, *C*, etc.), with the exception of single-qubit Pauli operators being written in the form
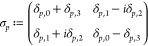
1for *p* ∈ {0, 1, 2, 3}. Sets are denoted by script letters  and vector spaces by bold script typeface . The state space of *N* qubits may be identified with the 2^*N*^ dimensional Hilbert space , with the space of (bounded) linear operators acting upon  denoted .

We introduce the *Pauli group*, , consisting of operators  for *p*_*i*_ ∈ {0, 1, 2, 3}, up to multiplication by ±1, ±*i*. Note the distinction between the bold font **σ** denoting tensor products and σ_*p*_ a single-qubit Pauli operator; we will sometimes write  to index explicitly the qubit position  on which it acts. We shall also make use of the *commutator* [*A*, *B*] ≔ *AB* – *BA* and *anticommutator* {*A*, *B*} ≔ *AB* + *BA*, defined for operators , which are zero when *A* and *B* commute/anticommute, respectively.

An *N*-qubit Hamiltonian can be written in the form
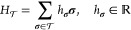
2for a set of Pauli operators ; specifying real coefficients ensures that  is *Hermitian*. The objective of quantum chemistry simulations is to estimate the ground state energy
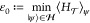
3where  is the expectation value of  with respect to some quantum state . Many physical properties of the target system are determined by the ground state, motivating this goal.

The Variational Quantum Eigensolver (VQE) quantum–classical hybrid algorithm^4^ is the most widely studied means of achieving this on NISQ hardware. VQE requires a parametrized ansatz state, , whose parameters **θ** are manipulated within a classical optimization scheme that aims to minimize the energy expectation value
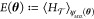
4evaluated via many prepare-and-measure cycles. The choice of ansatz restricts us to a subspace of quantum states and therefore must be carefully designed to be sufficiently expressible so as to capture the true ground state of the system.

A common form of ansatz state, particularly in relation to the electronic structure problem, is
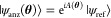
5where  is some fixed reference state in which the quantum circuit is initialized and  for parameters  and Pauli operators ; the unitary e^*iA*(**θ**)^ effects excitations above the reference state. Such ansätze as unitary coupled cluster (UCC)^13,14^ may by expressed by our choice of *A* (taking as reference the Hartree–Fock state), in addition to any others based on the theory of excitation operators such as ADAPT-VQE.^16−19^ The quantum advantage in VQE stems from the ability to prepare classically intractable states from our parametrized ansatz circuits.

## 3 Projections onto Stabilizer Subspaces

Given an operator , the space of quantum states  that it stabilizes are those satisfying **σ**|ψ⟩ = |ψ⟩, the +1-eigenspace of **σ**. Extending this notion to an Abelian subgroup of Pauli operators , there is an induced vector space  of states stabilized by the elements of .

A particularly useful definition is that of a Hamiltonian *symmetry*, taken here to mean a set  of Pauli operators such that
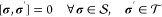
6In other words, a symmetry of  is any set of Pauli operators that commute universally among , which we may extend to an Abelian group , the closure of  under operator multiplication, which we shall call a *symmetry group*.

Note the setting in which we present symmetries here is stricter than the conventional definition, which considers any operator *S* that commutes with the Hamiltonian, i.e., , to be a symmetry. Such an operator need not commute with the individual terms as we require here. For example, in the Fermionic picture, the number operator  (where *a* is the Fermionic annihilation operator and its Hermitian conjugate *a*^†^ represents the creation operator) commutes with the full second-quantized molecular Hamiltonian, but not with an arbitrary excitation term.

The operators of  will in general be algebraically dependent, but the theory of stabilizers^50^ ensures the existence of a set of independent generators  such that . Now, recall that the Clifford group consists of unitary operators  (meaning ) with the property , i.e., *U normalizes* the Pauli group. We may construct a Clifford operation *U* mapping each symmetry generator to distinct single-qubit Pauli operators σ_*p*_, where we are free to choose *p* ∈ {1, 2, 3}. More precisely, there exists a subset of qubit positions  satisfying  and a bijective map  such that
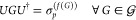
7

This is a powerful concept that provides a mechanism for reducing the number of qubits in the Hamiltonian while preserving its energy spectrum. This is at the core of qubit tapering,^47,48^ in which it is observed that
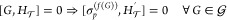
8implying the rotated Hamiltonian  consists solely of identity or Pauli σ_*p*_ operators in the qubit positions indexed by . Taking expectation values, one may replace the qubits  by their eigenvalues ν_*i*_ = ±1; each assignment
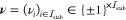
9defines a symmetry *sector* and at least one such sector will contain the true solution to the eigenvalue problem. Note the other sectors still have physical significance and may, for example, relate to solutions with different particle numbers or to excited states. Ancillary data files are provided in which we report the symmetry generators and corresponding sector for the Hamiltonians representing the molecular systems listed in Table 1.

**Table 1:** Systems Investigated to Benchmark the Noncontextual Projection Ansatz^a^

molecular systems			number of qubits		
name	charge	mult.	full	taper	CS-VQE^b^
Be	0	1	10	5	3
B	0	2	10	5	3
LiH	0	1	12	8	4
BeH	+1	1	12	8	6
HF	0	1	12	8	4
BeH_2_	0	1	14	9	7
H_2_O	0	1	14	10	7
F_2_	0	1	20	16	10
HCl	0	1	20	17	4

a All in the STO-3G basis.

b Indicates the fewest number of qubits required to achieve chemical precision.

A quantum state consistent with any such sector must be stabilized by the operators , and we may define a projection onto the corresponding stabilizer subspace. In general, a projection is defined to be an *idempotent* operator , i.e., *P*^2^ = *P*; the projection onto the ±1-eigenspace of a single-qubit Pauli operator σ_*p*_ for *p* ∈ {1, 2, 3} may be written
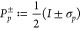
10States with no component inside the chosen eigenspace are mapped to zero and observe that
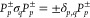
11for *q* ∈ {1, 2, 3}.

Let  be the reduced Hilbert space supported by the stabilized qubits  and  its complement such that . Given an assignment of eigenvalues , we may project onto the corresponding sector via
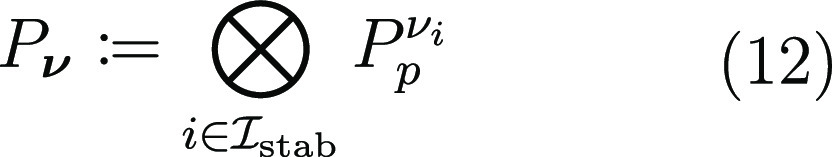
12and subsequently perform a *partial trace* over the stabilized qubits . This is effected by the unique linear map  satisfying the property  for all  and .

Finally, we may define the full stabilizer subspace projection map
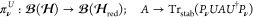
13which, using the linearity of Tr_stab_, yields a reduced Hamiltonian
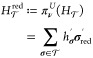
14where  and we have written . The new coefficients  differ from *h*_**σ**_ by a sign dependent on the chosen symmetry sector.

In qubit tapering, *U* is taken as eq 7 with the corresponding basis  a generating set for a full Hamiltonian symmetry.^47,48^ Assuming identification of the correct sector, the ground state energy of the -qubit reduced Hamiltonian  will coincide with the true value of the full system .

This stabilizer projection procedure is straightforward with respect to the Hamiltonian, since the stabilized qubits contain only operators with nonzero image under conjugation with *P*_**ν**_. However, suppose we were to take another observable  and wish to determine a reduced form on  that is consistent with the reduced Hamiltonian . This may be achieved by following precisely the same process that was applied to , but the symmetry  will not in general be a symmetry of *A* and therefore the “symmetry-breaking” terms (those which anticommute with the generators ) will vanish under projection onto the stabilizer subspace, as per eq 11. Letting  be the set of terms in the Pauli-basis expansion of *A*, observe that
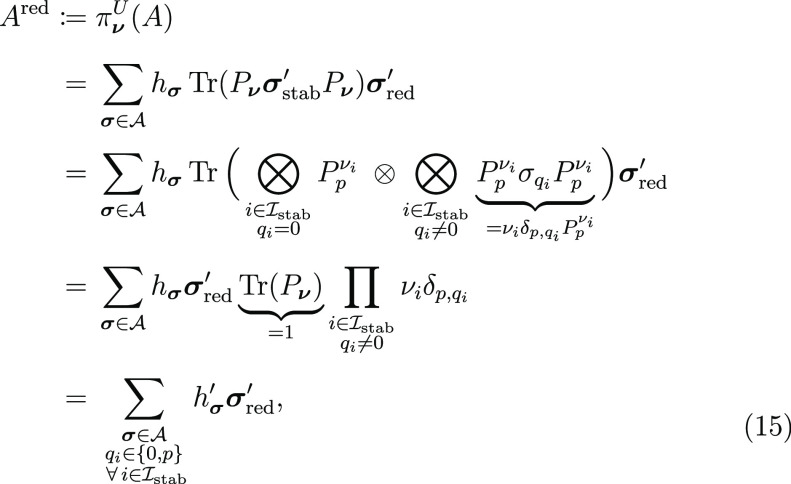
15recalling that *q*_*i*_ indicates the type of single-qubit Pauli acting on qubit position  in some tensor product **σ**, defined in Section 2.

The resulting form is identical to eq 14, except we are explicit that the terms surviving projection are only those whose qubit positions indexed by  consist exclusively of identity and Pauli σ_*p*_ operators; this is trivially true for the Hamiltonian by construction. Most importantly, this extends the stabilizer subspace projection to ansätze defined on the full system for use in variational algorithms. It should be noted that the above operations are classically tractable and can be implemented efficiently in the symplectic representation of Pauli operators.^51,52^

It would be remiss of us not to draw attention to the likeness of eq 13 to Positive Operator-Valued Measures (POVMs);^53^ indeed, the projectors (eq 12) define a complete set of *Kraus* operators.^54^ The stabilizer subspace projection procedure is reduced to a matter of enforcing a partial measurement over some subsystem of the full problem, for which the relevant outcomes have been determined via an auxiliary method. For example, this could involve identifying a quantum state with a known nonzero overlap with the true ground state; measuring the symmetry generators  in this state will yield the correct sector.

Hartree–Fock often provides such a state for electronic structure problems, although it is not immune to failure; this is particularly true in the strongly correlated regime. In these cases, we should defer to more effective reference states such as those obtained from Møller–Plesset perturbation theory (MP), coupled-cluster (CC) methods, and so on. One can imagine a hierarchy of increasingly precise ground state approximations, for which we should hope to obtain at some point a nonzero overlap with the true ground state.

## 4 CS-VQE in the Stabilizer Formalism

We now describe the *Contextual Subspace VQE* (CS-VQE) method in the stabilizer setting introduced in Section 3. CS-VQE partitions the Hamiltonian (2) into two disjoint components, one that is noncontextual and another that is contextual, which provides quantum corrections to the former via VQE.^45^ Explicitly, this allows us to write
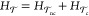
16where  is a noncontextual set of Pauli operators and  is what remains, which will in general be contextual.

CS-VQE differs from qubit tapering (described in Section 3) in the following way: the latter exploits existing (i.e., physical) symmetries of the Hamiltonian, whereas in CS-VQE, we impose additional “pseudosymmetries” derived from the noncontextual Hamiltonian. This results in a loss of information, since any terms of  not commuting with the symmetry generators will vanish under projection.

### 4.1 The Noncontextual Problem

The notion of contextuality goes back to the Bell–Kochen–Specker theorem.^55−57^ Here we use an explicit condition for the noncontextuality of a set of Pauli operators, developed by Kirby and Love^58^ and independently by Raussendorf et al.^59^ Strictly speaking, this condition tests for strong measurement contextuality. In this setting, a set  is understood to be noncontextual if and only if commutation forms an equivalence relation on , where we have defined the sub-Hamiltonian symmetry . There is an implied structure
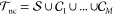
17where the  are equivalence classes with respect to commutation–in other words, elements of the same class commute and across classes they anticommute. Conversely, such a set of Pauli operators is contextual if and only if commutation fails to be transitive on .

The symmetry  can be expanded by taking pairwise products within equivalence classes, since {*C*_*i*_, *C*_*j*_} = 0 for  with *i* ≠ *j*, it is the case that  and we may define . As before, in Section 3,  induces a symmetry group for which one may define independent generators  and a Clifford operation  mapping the generators to single-qubit Pauli operators; the expectation value over these qubits will again be determined by an assignment  of eigenvalues, analogous to the selection of a symmetry sector in qubit tapering.

From each equivalence class , we select a representative *C*_*i*_ and construct an observable  where  and |**r**| = 1. Kirby and Love^60^ found that quantum states  stabilized by the operators  are consistent with a classical objective function η(**ν**, **r**) (derived in the Supporting Information), in the sense that η(**ν**, **r**) coincides with the noncontextual energy expectation value  for all parametrizations (**ν**, **r**). This is a consequence of the joint probability distribution chosen over the phase-space points of their (epistricted) model.^60,61^

The noncontextual energy spectrum is therefore parametrized by two vectors: the ±1 eigenvalue assignments **ν**, determining the contribution of the universally commuting terms, and **r**, encapsulating the remaining pairwise anticommuting classes. In this sense, we may refer to (**ν**, **r**) as a state of the noncontextual Hamiltonian itself, abstracted from quantum states of the corresponding stabilizer subspace. Optimizing over these parameters, we obtain the noncontextual ground state energy
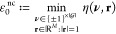
18and call an element (**ν**, **r**) of the preimage  a noncontextual ground state of . Let us denote by  the absolute error with respect to the true ground state energy.

As a classical estimate to the ground state energy of the full Hamiltonian , in Section 5, we found the difference between the noncontextual ground state and Hartree–Fock energy to be negligible for each of the molecules simulated, since the heuristic used to choose  prioritizes diagonal Hamiltonian terms. In principle, it may be an improvement upon Hartree–Fock as the noncontextual set can also take into account an off-diagonal contribution within the anticommuting classes. This is highly dependent on the chosen form of noncontextual set; a reformulation in terms of graphs, e.g., representing Pauli operators as nodes with (non)adjacency indicating (anti)commutation, will allow one to identify what the equivalent problem(s) are in computer science and therefore draw upon the vast body of existing research and select the best algorithms designed to solve such computational problems of graph theory. It should be noted that the “optimal” noncontextual subset will not necessarily be that which minimizes the noncontextual ground state energy and some consideration of the resulting quantum corrections must inform this choice, which remains an open question.

### 4.2 Quantum Corrections

Our simulation approach has thus far been strictly classical–now we arrive at the quantum element of CS-VQE. We have derived a classical estimate of the ground state energy from the noncontextual part of the Hamiltonian ; however, the contextual component  has so far been neglected.

While *C*(**r**) is not a stabilizer in the strict sense (it is not an element of the Pauli group), it is unitarily equivalent to one as a linear combination of anticommuting Pauli elements. Similar to the symmetry generators , it is possible to define a unitary operation *U*_*C*_ mapping *C*(**r**) onto a single-qubit Pauli operator, following the approach of unitary partitioning.^32−36^ However, unlike the  rotation,  is not Clifford as it collapses *M* terms onto a single Pauli operator and can therefore introduce additional terms to the Hamiltonian. Kirby et al.^45^ cautioned that, in principle, this increase in Hamiltonian complexity could be exponential in the number of equivalence classes *M*, namely, a scaling of . However, Ralli et al.^36^ demonstrated that the general scaling for this sequence of rotations (SeqRot) method is  where *x* ∈ [1, 2]; that is, still exponential, yet the necessary conditions to obtain the worst-case *x* = 2 are contrived and have not been observed for any molecular Hamiltonians investigated to date. Regardless, one may circumvent this potentially adverse scaling entirely by implementing the linear combination of unitaries (LCU) approach to unitary partitioning,^33,35^ which is only quadratic in the number of equivalence classes .^36^

Appending *C*(**r**) to our set of generators  and defining , there exists a subset of qubit indices  satisfying  and a bijective map  such that  for each . We reiterate that *p* ∈ {1, 2, 3} may be chosen at will; the approach taken by Kirby et al.^45^ is to select *p* = 3 to enforce diagonal generators.

Suppose we have a quantum state |ψ_(**ν**,**r**)_⟩ that is consistent with ; since the rotated state  must be stabilized by , the qubit positions  must be fixed. This implies a decomposition
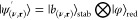
19where |*b*_(**ν**,**r**)_⟩ represents a single basis state of  and  is independent of the parameters (**ν**, **r**). The expectation value of the full Hamiltonian may be expressed as
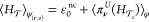
20where  contains only the terms of the contextual Hamiltonian that commute with all the noncontextual generators, just as in eq 15. It was observed by Kirby et al.^45^ that any term which anticommutes with at least one noncontextual generator must have zero expectation value, and our stabilizer subspace projection captures this fact.

Inspecting eq 20, we may optimize freely over quantum states φ, i.e., we are not constrained by the noncontextual ground state within . In fact, we may absorb the noncontextual ground state energy into the reduced contextual Hamiltonian
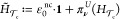
21defining the *contextual subspace Hamiltonian*; this form is obtained naturally when applying the stabilizer subspace projection to the full Hamiltonian, which automatically includes the noncontextual energy by fixing the corresponding eigenvalue assignments.

Now, we may perform unconstrained VQE to obtain a quantum-corrected estimate
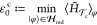
22of the true ground state energy with absolute error . We have equality when the stabilizers span every qubit position, which is the case when  since the generators must be algebraically independent: this means the initial quantum correction is trivial as the noncontextual part determines the entire system.

For instances of the electronic structure problem, there is no guarantee that  will achieve chemical precision (Δ_c_ < 1.6 mHa ≈ 4 kJ/mol) and, indeed, it might not improve upon the noncontextual estimate (although it will never be worse, due to the variational principle applying in this case). However, one can easily define a subset of  that is again noncontextual; this is achieved by discarding one of the noncontextual generators , along with the operators that it generates. We now append the discarded operators to the contextual Hamiltonian, relaxing the stabilizer constraint on the qubit position *f*(*G*) and permitting a search over its Hilbert space. This process may be iterated until the noncontextual set is exhausted and we recover full VQE. This means that, unless the ground state energy of  and *H* coincides, CS-VQE will improve upon the noncontextual energy using less quantum resources than full VQE; this is more rigorously defined in the next section.

In summary, what we have described here is a technique of scaling the relative sizes of the noncontextual (read classical) and contextual (read quantum) simulations in a reciprocal manner. We can therefore trade-off quantum and classical workloads in CS-VQE.

### 4.3 Expanding the Contextual Subspace

Now we describe the process of growing the contextual subspace more rigorously. We select a subset of noncontextual generators  whose stabilizer constraints we mean to enforce and construct a new noncontextual set ; the contextual set is expanded accordingly by appending the terms not generated by , i.e., . As before, there exists a unitary operation , a subset of qubit indices , and a bijective map  satisfying  (the rotation  may or may not be Clifford depending on whether *C*(**r**) is among the stabilizers we wish to fix).

Denote by  the ground state energy of the new noncontextual Hamiltonian  with absolute error . While this is weaker as an estimate of the true ground state energy of the full system, at the very least we are guaranteed to recover the initial noncontextual ground state energy from performing a simulation of the expanded contextual subspace,^45^ which we describe below.

The stabilizer constraints of  are enforced over the Hilbert space  of qubits indexed by , whereas we may perform a VQE simulation over , the Hilbert space of the remaining  qubits indexed by . Invoking the stabilizer subspace projection map  with the eigenvalue assignments  yields an expanded contextual subspace Hamiltonian
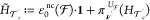
23Performing an -qubit VQE simulation over the contextual subspace, we obtain a new quantum-corrected estimate
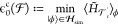
24with an error satisfying . Recall that  corresponds with the contextual error when we enforce the full set of noncontextual stabilizers.

Observe that, when , we are simply performing full VQE over the entire system; this occurs when we do not enforce the stabilizer constraint for any of the noncontextual generators, i.e., . Therefore, it must be the case that
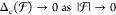
25Furthermore, given a nested sequence of generator subsets  with , then  and the convergence is monotonic. In this way, CS-VQE describes an interpolation between a purely classical estimate of the ground state energy and a full VQE simulation of the Hamiltonian. In the context of electronic structure calculations, this often permits one to achieve chemical precision at a saving of qubit resources, as indicated by Kirby et al.^45^ for a suite of tapered test molecules of up to 18 qubits. We note in eq 25 that the quality of the chosen ansatz and optimization procedure will limit the actual error one may achieve in practice. This statement instead indicates that, for an appropriate level of contextual subspace approximation, it is possible to construct a reduced Hamiltonian whose exact ground state lies within some error threshold of the true value.

Suppose we wish to find the optimal contextual subspace Hamiltonian of size *N*′ < *N*. The problem reduces to minimizing the error  over the  generator subsets  satisfying . CS-VQE is highly sensitive to this choice and remains a vital open question for the continued success of the technique. For chemistry applications, we grow the contextual subspace until the CS-VQE error attains chemical precision, which means finding the minimal  such that  < 1.6 mHa. In general, we will not have access to a target energy and so will not necessarily know when the desired precision is achieved; instead, we might choose the largest contextual subspace accommodated by the available quantum resource or iterate until the VQE convergence is within some fixed bound.

Greedily selecting combinations of *d* ≤ *N* generators that yield the greatest reduction in error is an effective stabilizer relaxation ordering heuristic, where iterating *k* < *N*/*d* involves a search of depth *d* over *N*–*dk* elements, thus necessitating  CS-VQE simulations. Taking *d* = 2 produces a good balance between efficiency and efficacy,^45^ but there is room for more targeted approaches that exploit some structure of the underlying problem. For example, in quantum chemistry problems, it could be that one should relax the stabilizers that have nontrivial action near the Fermi level, between the highest occupied molecular orbital (HOMO) and lowest unoccupied molecular orbital (LUMO). Excitations clustered around this gap are more likely to appear in the true ground state and should therefore not be assigned definite values under the noncontextual projection. This idea comes from the theory of pseudopotential approximations,^62^ in which it is observed that chemically relevant electrons are predominantly those of the valence space, whereas the core may be “frozen”, thus reducing the electronic complexity.

Alternatively, one might define a Hamiltonian term-importance metric that considers coefficient magnitudes^63^ or second-order response with respect to a perturbation of the Hartree–Fock state.^64^ In relation to this, it is also not clear which features of a molecular system mean that it might be more or less amenable to CS-VQE; additional insight here would allow one to predict how many qubits will be required to simulate a given problem to chemical precision.

It is not fully understood how CS-VQE relates to active space techniques more generally, but this would be an interesting pursuit for future work. For example, the downfolding technique of subsystem embedding subalgebra coupled cluster (SES-CC)^65^ presents a compelling approach that iteratively decouples excitations σ = σ_int_ + σ_ext_ into an “internal” part that belongs to a chosen excitation subalgebra and its “external” complement that may additionally be combined with the double unitary coupled cluster (DUCC) ansatz.^66^ This yields an effective Hamiltonian *H*_ext_^eff(DUCC)^ = (*P* + *Q*_int_) e^–σ_ext_^*H* e^σ_ext_^(*P* + *Q*_int_) where *P* projects onto the reference state and *Q*_int_ onto the subspace of excitations generated by σ_int_. This has a similar form to our stabilizer subspace projection (eq 13); indeed, it might be possible to reproduce SES-CC under a qubit mapping within the contextual subspace framework by identifying an appropriate noncontextual sub-Hamiltonian and stabilizer subspace.

A benchmark of this and other dimensionality reduction methodologies such as projection-based embedding (PBE)^67^ would be valuable. Furthermore, CS-VQE can be layered on top of these techniques to yield hybrid methods that might outperform any of them on their own; this is a consideration that we plan to take forward into further work, with the goal of deployment on larger molecular systems and basis sets.

### 4.4 The Noncontextual Projection Ansatz

CS-VQE has thus far not been applied to systems exceeding 18 qubits, and the resulting reduced Hamiltonians (eq b23) have been solved by direct diagonalization;^45^ clearly, this will not scale to larger systems, with the required classical memory increasing exponentially. Instead, they must be simulated by performing VQE routines, but defining an ansatz for the contextual subspace provided an obstacle to achieving this in practice.

However, having now placed the problem within the stabilizer formalism described in Section 3, we have already introduced (in Sections 4.1–4.3) the tools necessary to restrict an ansatz of the form in eq 5–defined over the full system–to the contextual subspace (eq 23). The approach adopted here is equivalent to that which we defined for qubit tapering in eq 15. To restrict a parametrized ansatz operator
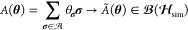
26in line with the stabilizer constraints , we may simply call upon the stabilizer subspace projection map  once more, which yields a restricted ansatz state
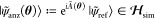
27where
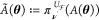
28Any rotated ansatz term  that is not identity or a Pauli σ_*p*_ on some subset of the qubit positions indexed by  will vanish.

The restricted reference state  is obtained from an effective partial projective measurement of  (see the discussion on POVMs in Section 3) with outcomes defined by **ν**′, which yields a product state
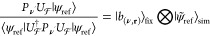
29where we have explicitly demarcated the separability across  and . The postmeasurement state  on the noncontextual subspace represents a single basis vector and can therefore be disregarded, leaving just the state of the contextual subspace; this we take as reference for our restricted ansatz. If the unitary partitioning rotations are *not* to be applied, then the  rotation is trivial over  and we incur no expense in coherent resource. However, if one does enforce the operator *C*(**r**) over the contextual subspace, there might be some nontrivial component of the rotation that must be applied in-circuit to ensure that the ansatz lies within the correct subspace; referring to Section 4.2, for the SeqRot approach this will consist of at most  CNOT operations in-circuit, whereas LCU is probabilistic due to the nature of block-encoding.^35^ Given a hardware-efficient ansatz, one may neglect this since the optimizer should compensate the parameters accordingly.

We may now define the contextual subspace energy expectation function
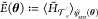
30with  as in eq 23, at which point we have reduced the problem to standard VQE, performed over a subspace of the full problem.

## 5 Simulation Results

The molecular systems that were simulated to benchmark the noncontextual projection ansatz for CS-VQE are given in Table 1. The molecule geometries were obtained from the Computational Chemistry Comparison and Benchmark Database (CCCBDB)^68^ and their Hamiltonians were constructed using IBM’s Qiskit Nature^69^ with PySCF as the underlying quantum chemistry package.^70^

Before we evaluate the efficacy of our noncontextual projection ansatz, there are a few features of eq 27 that should be highlighted. First of all, from the discussion following eq 29, we potentially apply some component of the operation  in-circuit, introducing further gates that will contribute additional noise. However, when the reference state is taken to be that of Hartree–Fock, we observed *Uψ*_ref_ to coincide with the noncontextual ground state. This is an artifact of the noncontextual set construction heuristic prioritizing diagonal entries, used within both this work and that of Kirby et al.^45^ This need not always be the case, but for the molecular systems investigated, this allows us to avoid performing  in-circuit and instead take the noncontextual ground state as our reference. Since we choose to rotate the noncontextual symmetry generators onto Pauli σ_3_ operators here, this may be prepared by applying a Pauli σ_1_ in each of the qubit positions  such that ν_*i*_ = −1 so that the corresponding reference state is stabilized by the relevant operators ν_*i*_σ_3_^(*i*)^. This is visible in Figure 2, in which the VQE routine is initiated with the optimization parameters zeroed, i.e., **θ** = **0**, and since e^*iÃ*(**0**)^ = **1**, optimization begins at the noncontextual ground state energy.

Second, application of the unitary partitioning rotations *U*_*C*_ to the ansatz operator *A*(**θ**) may introduce additional terms by a worst-case scaling factor of  where *M* is the number of equivalence classes in eq 17, although the true scaling is unlikely to be this severe as discussed in Section 4.2. We obtained M = 2 for all of the molecules tested, in which case SeqRot is identical to the asymptotically favorable LCU method. In fact, for small *M* ≪ *N*, SeqRot may generate fewer terms than LCU (Ralli et al. presented a toy problem with *M* = 3 in which this was the case^36^) and therefore our choice of SeqRot here is valid given that the noncontextual set  construction heuristic prioritizes the universally commuting terms  in eq 17. Different heuristics may lead to larger values for *M*, in which case, we recommend an adoption of LCU for implementations of CS-VQE.

**Figure 1:**
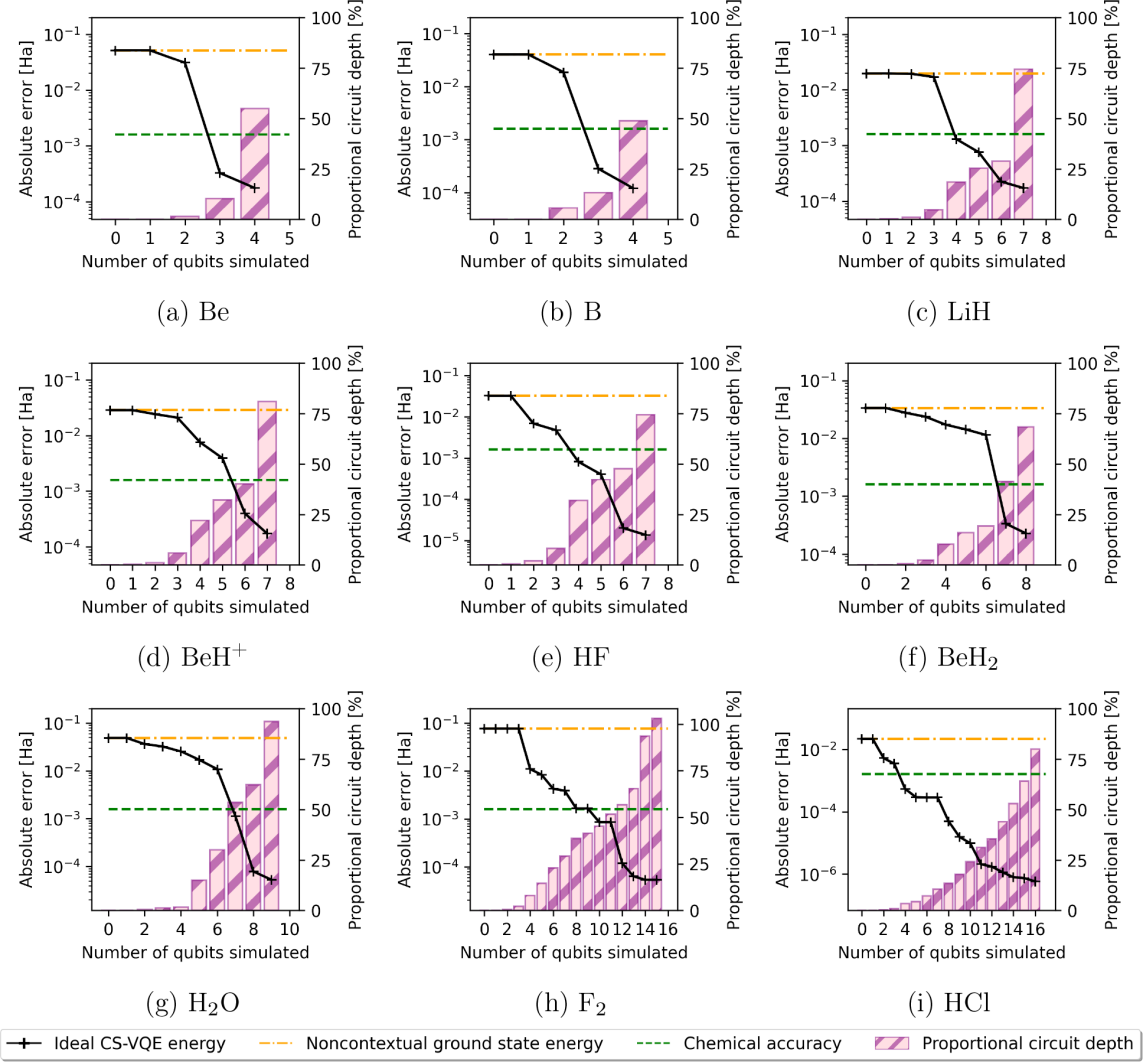
Ideal CS-VQE errors (left-hand axis) and corresponding noncontextual projection ansatz circuit depths as a proportion of the full UCCSD operator from which it is derived (right-hand axis) against the number of qubits simulated.

Despite this, upon the subsequent projection of *A*(**θ**), it is possible that a significant number of terms will vanish. This is highly dependent on the quality of the initial ansatz and how heavily it is supported on the stabilized qubit positions . Figure 1 presents circuit depths of the noncontextual projection ansatz as a proportion of the base ansatz from which it is derived, in this case the unitary coupled-cluster singles and doubles (UCCSD) operator. A net reduction in circuit depth is observed, which is quite dramatic up to the point of reaching chemical precision in the CS-VQE routine; in Table 2, we give the specific number of ansatz terms before and after application of the noncontextual projection to UCCSD and UCCSDT for the fewest number of qubits permitting chemical precision.

**Table 2:** Number of Pauli Terms  for a Selection of (Tapered) Ansätze^a^

		number of terms in ansatz operator		
molecule		UCCSDT (full/proj)^b^	UCCSD (full/proj)^b^	ADAPT-VQE^c^
Be	3	(48/6)	(48/6)	5
B	3	(48/12)	(32/4)	3
LiH	4	(704/53)	(192/53)	5
BeH^+^	6	(646/191)	(166/79)	11
HF	4	(92/57)	(92/57)	4
BeH_2_	7	(1312/352)	(224/96)	10
H_2_O	7	(1892/942)	(324/238)	21
F_2_	10	(176/114)	(176/114)	12
HCl	4	(348/40)	(348/40)	4

a Each simulation is plotted in Figure 2.

b The number of qubits in the contextual subspace over which the ansatz is projected; each tuple (full/proj) gives the number of terms pre- and postprojection.

c The number of ADAPT-VQE cycles required to achieve chemical precision, with the operator pool consisting of the projected UCCSD terms.

In order to identify a compact ansatz that closely captures the underlying chemistry with minimal redundancy, we employ the ADAPT-VQE methodology.^16−19^ The algorithm centers around an operator pool from which terms are selected in line with a gradient-based argument and appended to a dynamically expanding ansatz whose parameters are optimized at each cycle via VQE. The particular approach we implement here is that of qubit-ADAPT-VQE,^17^ following on from iterative qubit coupled cluster,^71^ which searches at the level of Jordan–Wigner encoded Pauli operators; the seminal ADAPT-VQE paper^16^ instead defines its operator pool over Fermionic excitations.

The Jordan–Wigner transformation^72^ maps a single Fermionic annihilation operator onto two Pauli operators
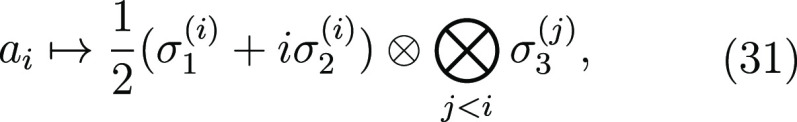
31with the creation operator given by its Hermitian conjugate *a*_*i*_^†^. Therefore, an excitation on  spin orbitals of the form
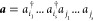
32is represented by 2^2*s*^ Pauli operators under this encoding. In the unitary coupled cluster theory, we are interested rather in the operator ***a***–***a***^†^ to ensure unitarity upon exponentiation; this may be expressed by 2^2*s*–1^ Pauli terms.

As such, after a mapping onto qubits via the Jordan–Wigner transformation, single, double, and triple excitations account for 2, 8, and 32 Pauli operator terms, respectively; while these are required to enforce various electronic symmetries in the ansatz state, not all are necessary to reach chemical precision. This idea lies behind qubit-ADAPT-VQE, which will select only the necessary Pauli terms and therefore yields considerably reduced circuit depths.^17^

To leverage ADAPT-VQE in the context of CS-VQE, we define an operator pool  and apply to it the stabilizer subspace projection (eq 13) to define a reduced pool  for the corresponding contextual subspace. Projecting the full pool in this way will ensure that any symmetries *S* present will be preserved, since , allowing us to incorporate some chemical intuition into the contextual subspace despite an abstraction from the original problem; one could define a reduced pool directly, but care should be taken to avoid the inclusion of symmetry-breaking terms that may needlessly increase the complexity of the ADAPT-VQE procedure. The algorithm is then executed as normal, only terminating once the ADAPT-VQE energy is chemically precise with respect to the FCI energy; for scalability, one should terminate computation when the largest gradient in magnitude falls below some predefined threshold, since the true ground state energy will not in general be known. In the Supporting Information, we provide a detailed description of the specific ADAPT-VQE implementation used within this work.

For the following, we take our pool  to be the terms of the UCCSD operator for each of the molecules in Table 1 before tapering and projecting into the relevant contextual subspace. In Figure 2, we present the ADAPT-VQE convergence data with expectation values obtained via exact wave function (statevector) calculations (i.e., no statistical/hardware noise); chemical precision is achieved in each instance. We used the adaptive moment estimation (Adam)^73^ classical optimizer and computed parameter gradients as per the parameter shift rule.^74^ Adam has been adopted for previous research in VQE for its resilience to noise, although it exhibits relatively slow convergence compared with other optimizers^75,76^ such as Broyden–Fletcher–Goldfarb–Shanno (BFGS)^77^ and quantum natural gradient (NatGrad);^78^ the latter might be preferable for future work.

**Figure 2:**
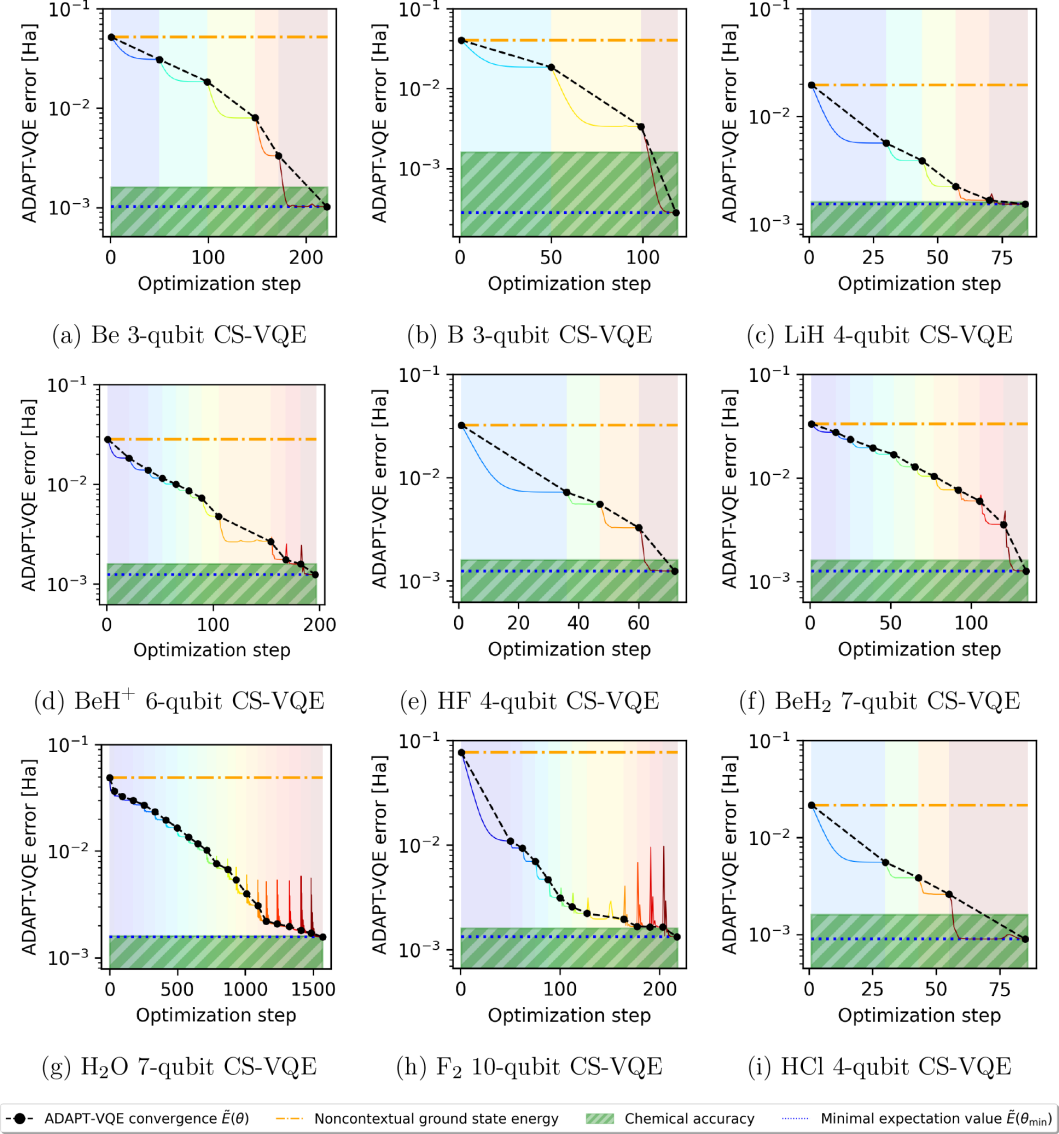
Validation of the noncontextual projection approach to ansatz construction for CS-VQE (eq 27), used here in conjunction with ADAPT-VQE.^16−19^ We plot (on a log_10_ scale) the absolute error of wave function simulations conducted for the suite of trial molecules outlined in Table 1, each shown to achieve chemical precision; the horizontal axis indicates the algorithm step counter with each shaded region a separate ADAPT-VQE cycle. Adaptive moment estimation (Adam)^73^ is the classical optimizer taken in the VQE routine performed over the contextual subspace for each ADAPT-VQE cycle, and the settings used are as follows: tolerance = 10^–4^, learning rate = 10^–2^, β_1_ = 0.4, β_2_ = 0.999, ϵ = 10^–8^. The parameter gradients ∂*Ẽ*(**θ**)/∂θ_*i*_, required for both operator pool term selection and VQE, were computed using the parameter shift rule.^74^

The number of ADAPT-VQE cycles (and therefore the number of terms in the resulting ansatz operator) are presented in Table 2, alongside the size of the projected UCCSD operator pool used; one observes a significant reduction in the number of terms. The optimized ADAPT-VQE ansatz operators are reported in ancillary data files, along with a description of the smallest CS-VQE problem permitting chemical precision. This includes the optimal noncontextual generator subset , the resulting noncontextual projection ansatz (eq 27), the restricted reference state  (eq 29), the target error  (eq 25), and that which was actually achieved in our VQE simulations (Figure 2). We also include the corresponding contextual subspace Hamiltonians for reproducibility.

Extracting the optimal parameter configuration **θ**_min_ from the wave function simulations in Figure 2, we subsequently assess the effect of sampling noise on the simulation error with our ansatz circuit preparing the optimal quantum state . Note that, for each of the molecular systems in Table 1, **θ**_min_ is given explicitly in the ancillary data files.

To achieve an absolute error of Δ > 0, one should expect to perform  shots (for each term of the Hamiltonian).^4^ Conversely, suppose we are allocated a quantity  of shots; the obtained error should be of the order . In order to increase estimate accuracy, we collected the Pauli terms into qubit-wise commuting (QWC) groups^25^ using the graph-coloring functionality of NetworkX;^79^ such groups may be measured simultaneously.

**Figure 3:**
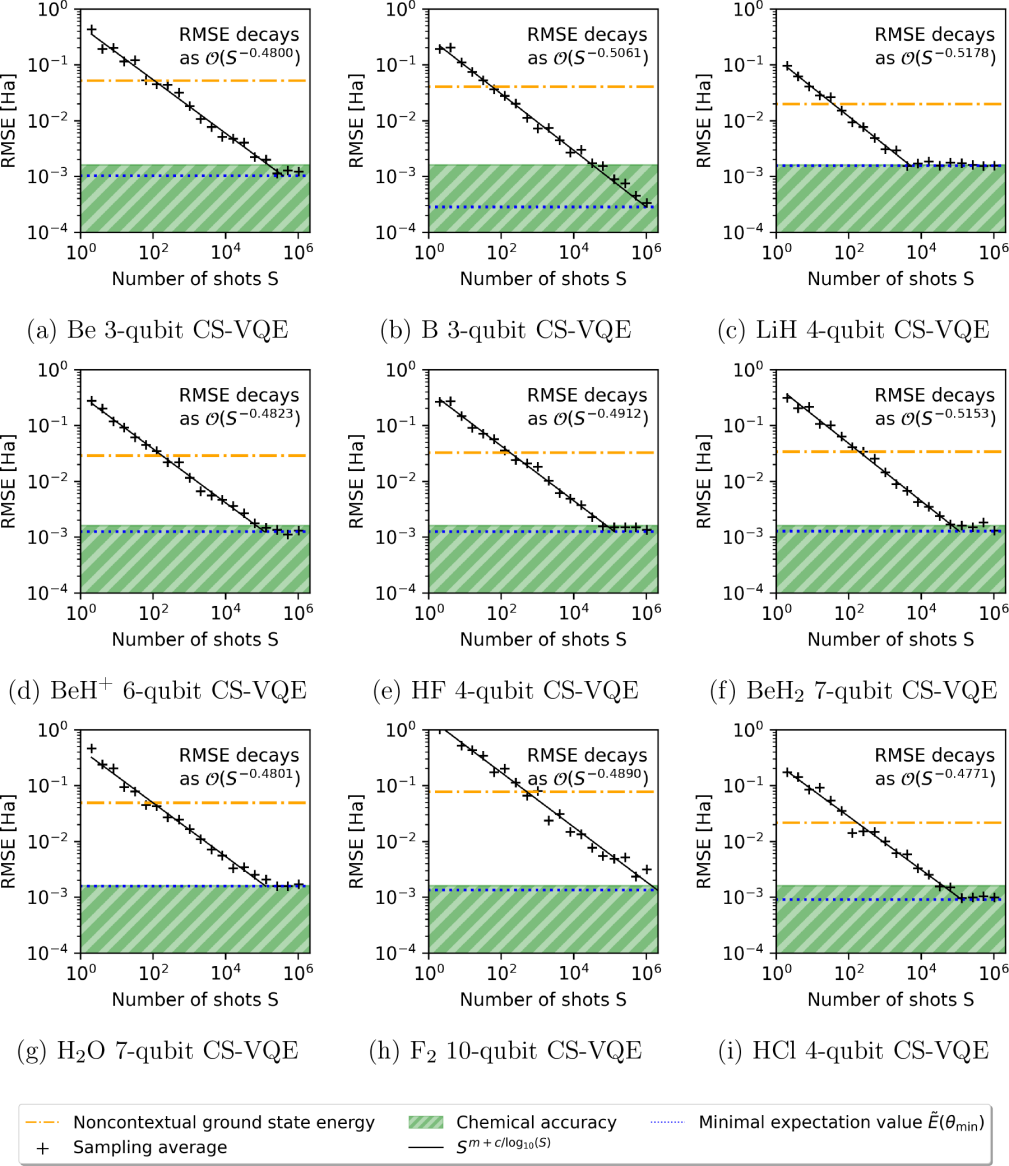
Each of the plots in panels a–i correspond with Figure 2a–i and illustrate the statistical effect of sampling noise at the optimal parametrization **θ**_min_ determined from the ADAPT-VQE statevector simulations in Figure 2. We plot the root-mean-square error (RMSE) for 20 “realizations” of the ground state energy estimate with *S* ≤ 2^20^ shots executed via IBM’s QASM simulator; determining the line of best fit *m*·log_10_(*S*) + *c* with respect to the log–log data indicates a decay in error of .

In Figure 3, the number of shots *S* = 2^*n*^ for *n* = 0, ..., 20 carried out per QWC group is varied, and we observe the root mean-square error (RMSE) over 20 realizations of the ground state energy estimate, plotted on a log–log scale. For clarity, note the **only** source of noise here is that which arises from statistical variation of the quantum circuit sampling; we have **not** introduced hardware noise in the form of imperfect quantum gates or decoherence.

Two error regimes are observed, one of which is quite trivial: at high shot counts, we see a plateau resulting from the optimal error  being recovered. To assess the convergence properties outside of this limiting region, we plot a line of best fit *m*·log_10_(*S*) + *c* among the data not exhibiting such behavior; since the data is represented on a log–log scale, this corresponds with a decay in error of . In each plot of Figure 3, we obtain *m* ≈ −0.5, meaning the RMSE follows the predicted decay of .

In every simulation bar F_2_, chemical precision was achieved within *S* = 2^20^ ≈ 10^6^ shots per QWC group. However, our shot budget could be reduced by implementing more advanced allocation strategies, for example, according to the magnitude of Hamiltonian term coefficients^80^ or a classical shadow tomography approach.^30,31^

## 6 Conclusions

We have placed CS-VQE on the theoretical footing of stabilizer subspace projections, which allows one to compare it against other qubit reduction techniques such as qubit tapering.^47,48^ Tapering defines a projection dependent on a symmetry of the full Hamiltonian and preserves the ground state energy exactly, whereas CS-VQE is approximate and projects onto a contextual subspace consistent with the symmetry of the noncontextual sub-Hamiltonian, augmented by an anticommuting contribution. In combination, the two techniques can effect a significant reduction in quantum resource requirements, as illustrated by Kirby et al.^45^ and in Figure 1.

Previously, the only obstacle to building a CS-VQE framework that would be faithful to deployment on quantum devices was that of the ansatz, which has been addressed within this work. Furthermore, we demonstrated how CS-VQE may be combined with the ADAPT-VQE^16−19^ ansatz construction framework by applying our noncontextual projection to the operator pool; validation was presented in Figure 2 in which we achieved chemical precision for the suite of small molecules outlined in Table 1. This combination provides considerable flexibility in both qubit count and circuit depth, allowing one to identify a reduced problem that may be simulated on the available quantum resource.

A number of research questions concerning the scalability of CS-VQE remain; we recapitulate these here. First, the success of CS-VQE is sensitive to the generator subset  one chooses to constrain in the stabilizer subspace projection. To date, the most effective method for choosing this subset has been a greedy-search heuristic necessitating  VQE simulations where *d* ≤ *N* is the search depth; this is expensive for NISQ hardware, and there is room for more targeted heuristics. For example, we may draw on chemical intuition to inform the selection of a contextual subspace that captures information about the underlying electronic structure problem. The second obstacle lies in the approach taken to construct the noncontextual sub-Hamiltonian. There is currently no intuition as to what constitutes an effective choice here, although it should be noted that the “optimal” noncontextual subset will not necessarily be that which minimizes the noncontextual ground state energy; some consideration of the resulting contextual subspaces must come into the construction of the noncontextual problem. We leave these issues for future work.

The natural next step is to execute this method on a NISQ computer, challenging the current best-in-class electronic structure simulations from Google, IonQ, and IBM.^1−3^ To achieve this goal, CS-VQE could be combined with techniques of measurement reduction^20−35^ and error mitigation.^37−44^

Finally, we have written an open-source Python package that facilitates the stabilizer subspace projection techniques of this work, with in-built tapering and CS-VQE functionality. We welcome the reader to make use of our code,^49^ which is freely available on GitHub.

## Data Availability

In the interest of reproducibility, we supply in the Supporting Information the tapering parameters, CS-VQE model data, and noncontextual projection ansätze which permit chemical precision for the fewest number of qubits with respect to the molecular systems listed in Table 1; the raw data for these results are supplied in ancillary files hosted at https://arxiv.org/abs/2204.02150 along with an explanatory notebook. This will provide sufficient information for the reader to reproduce Figures 1, 2 and 3.

## Supporting Information Available

Further information concerning circuit construction, deriving the noncontextual objective function one must solve to perform CS-VQE and a closer look at the ADAPT-VQE algorithm (PDF)
Tapering parameters, CS-VQE model data and noncontextual projection ansätze which permit chemical precision for the fewest number of qubits with respect to the molecular systems listed in Table 1—also includes the raw data required to reproduce Figures 1, 2 and 3, with an accompanying notebook (ZIP)
