# Supplementary material for: A Stabilizer Framework for the Contextual Subspace Variational Quantum Eigensolver and the Noncontextual Projection Ansatz
Source: J Chem Theory Comput. 2023 Jan 23;19(3):808–21. doi: 10.1021/acs.jctc.2c00910 (PMC9933439; doi:10.1021/acs.jctc.2c00910)
Supplement: Supplementary file 1 — ct2c00910_si_001.zip [file ct2c00910_si_001.zip › anc/data_access.html]

data\_access


# Data access for reproducibility¶

In this notebook we describe the data structure for the provided ancillary files. This allows the user to access Qubit Tapering and Contextual Subspace VQE model data, ADAPT-VQE simulations over the smallest contextual subspace permitting chemical accuracy and shot noise sampling for the following molecular systems:

In [1]:

```
speciename_list = [
    'Be_STO-3G_SINGLET',
    'B_STO-3G_DOUBLET',
    'LiH_STO-3G_SINGLET',
    'HF_STO-3G_SINGLET',
    'BeH+_STO-3G_SINGLET',
    'BeH2_STO-3G_SINGLET',
    'H2O_STO-3G_SINGLET',
    'F2_STO-3G_SINGLET',
    'HCl_STO-3G_SINGLET'
]
```

## Accessing the tapering/CS-VQE model data¶

For each of the molecules investigated in this paper the reader may access various tapering and CS-VQE parameters as follows:

In [2]:

```
import json

speciesname = 'Be_STO-3G_SINGLET'
with open('model_data/model_data_'+speciesname+'.json', 'r') as infile:
    model_data = json.load(infile)
```

With the file loaded, the data is structured like so:

## General¶

- `ham` contains the tapered Hamiltonian.
- `ansatz` contains a number of tapered an\"atze:
  - `uccs`
  - `uccsd`
  - `uccsdt`
  - `excitek` for $k \in \{2,3,4,5,6,7\}$ contains excitation terms whose coefficient exceeds a threshold $10^{-k}$
- `num_qubits` is the number of qubits in the *tapered* problem.

## Tapering parameters¶

- `num_tapered` is the number of qubits discarded through tapering, so num\_tapered + num\_qubits gives the number of qubits in the original problem.
- `symmetrygen` the Hamiltonian symmetry generators.
- `taperqubits` the qubit positions that the generators are rotated onto.
- `tapersector` the assignment of $\pm1$ eigenvalues the the symmetry generators, i.e. the symmetry sector.
- `stabilizers` the rotated generators, consisting of single-qubit Pauli $X$ operators.

## CS-VQE parameters¶

- `hf_config` the Hartree-Fock state stored as a binary string.
- `nc_config` the noncontextual ground state stored as a binary string.
- `terms_noncon` the noncontextual set identified when constructing the CS-VQE model.
- `ham_rotations` the stabilizer rotations mapping generators to single-qubit Pauli operators.
- `noncon` the noncontextual ground state energy.
- `hf_energy` the Hartree-Fock energy - coincides with `noncon`.
- `truegs` the true ground state energy of the full system.
- `G` the rotated symmetry generators, with their coefficients the optimal eigenvalue assignment $\vec{\nu}$.
- `A` the rotated anticommuting class operator $A(\vec{r})$, with the optimal $\vec{r}$ stored as its coefficients.
- `cs_vqe_energy` the CS-VQE energies for increasing contextual subspace dimension. The $i$-th element corresponds with an $i$-qubit subspace.
- `cs_vqe_errors` the CS-VQE errors for increasing contextual subspace dimension. The $i$-th element corresponds with an $i$-qubit subspace.
- `chem_acc_num_q` the fewest number of qubits permitting chemical accuracy ($<1.6$ mHa).
- `order` the stabilizer relaxation ordering.
- `ham_reduced` contextual subspace Hamiltonians, where the $i$-th element is an $i$-qubit Hamiltonian.
- `expressible` indicates whether the the ans\"atze in `ansatz` are sufficiently expressible.

In [3]:

```
model_data.keys()
```

Out[3]:

```
dict_keys(['ham', 'ansatze', 'num_qubits', 'hf_config', 'nc_config', 'terms_noncon', 'num_tapered', 'true_gs_nrg', 'ham_rotations', 'noncon', 'truegs', 'G', 'A', 'X_index', 'X_qubit', 'cs_vqe_energy', 'cs_vqe_errors', 'chem_acc_num_q', 'order', 'ham_reduced', 'expressible', 'symmetrygen', 'taperqubits', 'tapersector', 'stabilizers', 'hf_energy'])
```

## Accessing the ADAPT-VQE simulation data¶

We also include noiseless ADAPT-VQE and shot noise data for the molecules.

In [4]:

```
with open('simulation_data/ADAPT-VQE/ADAPT-VQE_results_'+speciesname+'.json', 'r') as infile:
    data = json.load(infile)
    
optimized_ansatz = data['optimized_ansatz']
adapt_vqe_interim_data = data['adapt_vqe_interim_data']
```

With the file loaded, the data is structured like so:

- `optimized_ansatz`

  - `optimal_energy` final converged ADAPT-VQE energy
  - `optimal_params` the ansatz parametrization yielding the above energy
  - `optimal_pool_indices` the indices of the UCCSD operator selected via ADAPT-VQE
  - `optimal_pool_labels` the Pauli operators in the final ADAPT-VQE ansatz
- `adapt_vqe_interim_data`

  - first level is the ADAPT cycle `1`, `2`, `3` etc.
    - `gradients` operator pool gradients at each ADAPT cycle
    - `optimal_pool_indices` growing list of the pool indices with largest gradient
    - `vqe_result` the result of the VQE simulation punctuating each ADAPT cycle
    - `interim_results` the interim VQE data
    - `termination_value` the value that dicatates whether ADAPT-VQE terminates - here it represents the VQE energy error with respect to the true ground state energy (terminating when it is below chemical accuracy, i.e. 0.0016) but in general it should be taken as the largest operator gradient at each cycle.
    - `termination_status` indicates whether ADAPT-VQE has converged

In [5]:

```
for key, value in optimized_ansatz.items():
    print(f'{key}: {value}')
```

```
optimal_energy: -14.40263546868186
optimal_params: [0.1100367118949897, 0.18572195133965905, 0.07657961833574932, 0.10999067535098805, 0.07664750028718734]
optimal_pool_indices: [2, 0, 5, 4, 3]
optimal_pool_labels: ['IYX', 'XXY', 'YZX', 'YIX', 'ZYX']
```

## Accessing the shot-noise sampling data:¶

In [6]:

```
with open('simulation_data/SNS/shot_noise_sampling_'+speciesname+'.json', 'r') as infile:
    sns_data = json.load(infile)
```

With the file loaded, the data is structured like so:

- First level indicates increasing numbers of shots, with the index $s$ in `1`, `2`, `3` etc.
  - `n_shots` the precise number of shots performed, given by $2^s$
  - `data` the energy output, averaged over samples
  - `raw_samples` the individual samples, allowing one to compute the root-mean-square error (RMSE)
  - `mit_samples` error mitigation not relevant since run using IBM's QASM simulator with no hardware noise

In [7]:

```
import numpy as np

for s,data in sns_data.items():
    n_shots = data['n_shots']
    samples = data['raw_samples']
    RMSE = np.sqrt(np.mean(np.square(np.array(samples)-model_data['truegs'])))
    print(f'RMSE for {n_shots: <7} shots is {RMSE}')
```

```
RMSE for 2       shots is 0.42822711881098036
RMSE for 4       shots is 0.19122442480164228
RMSE for 8       shots is 0.19783012440263054
RMSE for 16      shots is 0.11273086469973188
RMSE for 32      shots is 0.11984224920346417
RMSE for 64      shots is 0.05242376332528823
RMSE for 128     shots is 0.04481330422643451
RMSE for 256     shots is 0.04332333940147248
RMSE for 512     shots is 0.03130029710470042
RMSE for 1024    shots is 0.0179488295683144
RMSE for 2048    shots is 0.010694410131857027
RMSE for 4096    shots is 0.00763137382542235
RMSE for 8192    shots is 0.005022020605377695
RMSE for 16384   shots is 0.004659633184381179
RMSE for 32768   shots is 0.004001380193161569
RMSE for 65536   shots is 0.0022196656972968873
RMSE for 131072  shots is 0.001979115794101689
RMSE for 262144  shots is 0.0011259432575134124
RMSE for 524288  shots is 0.0012645622517898343
RMSE for 1048576 shots is 0.0012044075899128389
```

# Reproducing figures¶

We also provide plotting functions to reproduce the relevant figures:

In [8]:

```
from plotting import plot_adapt_vqe_results
fig = plot_adapt_vqe_results(adapt_vqe_interim_data, model_data)
```

In [9]:

```
from plotting import plot_sns_results
converged_energy = optimized_ansatz['optimal_energy']
fig = plot_sns_results(sns_data, model_data, converged_energy)
```
