# Supplementary material for: A Stabilizer Framework for the Contextual Subspace Variational Quantum Eigensolver and the Noncontextual Projection Ansatz
Source: J Chem Theory Comput. 2023 Jan 23;19(3):808–21. doi: 10.1021/acs.jctc.2c00910 (PMC9933439; doi:10.1021/acs.jctc.2c00910)
Supplement: Supplementary file 2 — ct2c00910_si_002.pdf [file ct2c00910_si_002.pdf]

Supporting Information: A Stabilizer Framework for Contextual Subspace VQE and the Noncontextual Projection Ansatz

Tim Weaving,[*,†] Alexis Ralli,[†] William M. Kirby,[‡] Andrew Tranter,[¶] Peter J. Love,[‡,§] and Peter V. Coveney[*,†,⊥]

†*Centre for Computational Science, Department of Chemistry, University College London, WC1H 0AJ, United Kingdom*

‡*Department of Physics and Astronomy, Tufts University, Medford, MA 02155, USA*

¶*Cambridge Quantum Computing, 9a Bridge Street Cambridge, CB2 1UB, United Kingdom*

§*Computational Science Initiative, Brookhaven National Laboratory, Upton, NY 11973, USA*

‖*UCL Centre for Advanced Research Computing, Gower Street, London WC1E 6BT, United Kingdom*

⊥*Informatics Institute, University of Amsterdam, Amsterdam, 1098 XH, Netherlands*

* E-mail: timothy.weaving.20@ucl.ac.uk; p.v.coveney@ucl.ac.uk

1 Circuit Implementation

Here we introduce the key concepts that are required to implement our CS-VQE ansatz as a quantum circuit, withholding a thorough introduction to the topic (the reader is referred to Nielsen and Chuang for this[1].

1.1 Trotterization

For operators $A, B \in \mathscr{B}(\mathscr{H})$, we have $e^{A+B} = e^A e^B$ if and only if A commutes with B (i.e. $[A, B] = 0$), contrary to the familiar rules of exponentiation for numbers; in the more general case, the Lie product formula states

$$e^{A+B} = \lim_{n\to\infty} \left(e^{A/n} e^{B/n}\right)^n. \tag{1}$$

The technique of *Suzuki-Trotter* truncates the above limit at some $n_\mathrm{T} \in \mathbb{N}$ (the *Trotter number*) to yield an approximation of the exponentiated operator sum. Given a sum of operators $\sum_k \theta_k A_k, \theta_k \in \mathbb{R}$, we may write the first-order Trotter expansion

$$e^{i \sum_k \theta_k A_k} \approx \left(\prod_k e^{i \frac{\theta_k}{n_\mathrm{T}} A_k} \right)^{n_\mathrm{T}}, \tag{2}$$

where the exponentiated Pauli operator $e^{i \frac{\theta_k}{n_\mathrm{T}} A_k}$ may be performed in-circuit as described in 1.2 using $\mathcal{O}(N)$ native quantum gates.

Used in combination with VQE, $n_\mathrm{T} = 1$ is often sufficient to achieve high levels of precision since we expect the optimizer to produce different coefficients that counteract the Trotter error. It has also been observed that the ordering of Trotter terms has an impact on errors[2] – this will not be explored here, however it is a possible consideration for future research. There is contention over Trotterized UCC and whether it is well-defined as an ansatz, since the ordering of terms can induce variations in error beyond chemical accuracy, particularly for strongly correlated systems[3].

1.2 Exponentiating Pauli Strings

Given a Pauli operator $P \in \mathcal{P}_N$ and some angle $\theta \in \mathbb{R}$, we would like to implement the exponential $e^{i\theta P}$ as a quantum circuit; this can be achieved with $\mathcal{O}(N)$ gates. We shall first assume that P consists of just Pauli I, Z operators, with the qubit positions acted upon by Z indexed with the set $\mathcal{I}_Z$. Observe that

$$\begin{aligned} e^{i\theta Z^{\otimes \mathcal{I}_Z}} |\boldsymbol{b}\rangle &= \Big(\cos\theta + i(-1)^{\rho_{\mathcal{I}_Z}(\boldsymbol{b})}\sin\theta\Big)|\boldsymbol{b}\rangle \\ &= e^{i\theta(-1)^{\rho_{\mathcal{I}_Z}(\boldsymbol{b})}}|\boldsymbol{b}\rangle . \end{aligned} \tag{3}$$

where we have omitted the qubit positions that are identity. Therefore, we may realise this operation by storing $\rho_{\mathcal{I}_Z}(\boldsymbol{b})$, the parity of $|\boldsymbol{b}\rangle$ over $\mathcal{I}_Z$, in one of the qubits and applying to it an R_Z gate, defined by

$$R_Z(\phi) = \begin{pmatrix} e^{i\phi/2} & 0 \\ 0 & e^{-i\phi/2} \end{pmatrix}. \tag{4}$$

In (3), even parity results in a phase $e^{i\theta}$ whereas odd parity yields $e^{-i\theta}$ – these are obtained by application of $R_Z(2\theta)$ to each of $|0\rangle, |1\rangle$, respectively. The parity computation is accomplished via a ‘cascade’ of CNOT gates between adjacent qubits. We are now in a position to explicitly write down a quantum circuit that effects the exponentiation of a Pauli string consisting of I, Z operators, presented in Figure 1.

This is the basic building block for exponentiating an arbitrary Pauli string, as we are

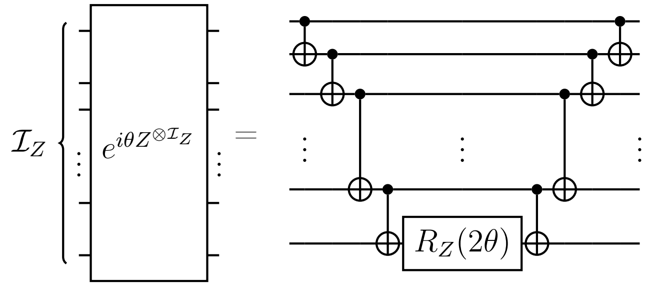

Figure 1: Circuit to realise the exponential $e^{i\theta P}$ where P consists of just Pauli I, Z operators. The qubits set to identity are omitted from the diagram.

able to make a change of basis from Pauli X, Y operators to a Pauli Z. Note that $X = HZH$ and $Y = SHZHS^\dagger$ where $S = R_Z(\pi/2)$. Once this transformation has been applied, the problem is reduced to that of before and we perform the same parity computation and Z rotation. We end with the reverse transformation taking us back into the original basis – the complete circuit is presented in Figure 2. The total number of gates is at most $6N - 1$, achieved only when P consists solely of Pauli Y operators acting upon all of the N qubit positions.

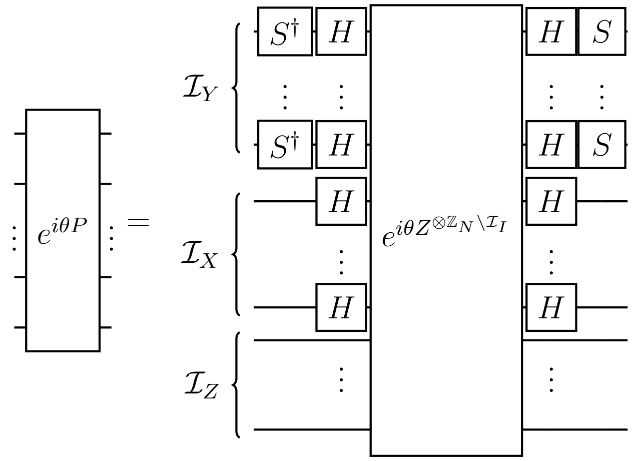

Figure 2: Circuit to realise the exponential $e^{i\theta P}$ where P is an arbitrary Pauli string. The qubits set to identity are omitted from the diagram.

It is noted that in the Trotterized circuit there will be many blocks of exponentiated Pauli strings of the form displayed in Figure 2, and it is possible that gate cancellation will occur between adjacent blocks where we have $SS^\dagger = H^2 = \mathbb{1}$. Furthermore, the implementation presented here is not unique – different circuits yielding the same quantum states are possible.

2 Noncontextual Objective Function

The CS-VQE method relies on solving a classical objective function that defines the energy spectrum of the noncontextual Hamiltonian

$$H_{\mathcal{T}_{\text{nc}}} = \sum_{P \in \mathcal{T}_{\text{nc}}} h_P P. \tag{5}$$

The objective function is constructed from the decomposition

$$\mathcal{T}_{\text{nc}} = \mathcal{S} \cup \mathcal{C}_1 \cup \cdots \cup \mathcal{C}_M, \tag{6}$$

in which one observes that the sum over $\mathcal{T}_{\text{nc}}$ may be separated into individual summations over the symmetry terms $\mathcal{S}$ and the anticommuting classes $\mathcal{C}_i$ for $i \in \{1, \dots, M\}$, i.e. $\sum_{P \in \mathcal{T}_{\text{nc}}} = \sum_{P \in \mathcal{S}} + \sum_{i=1}^{M} \sum_{P \in \mathcal{C}_i}$. Furthermore, since $\mathcal{G}$ generates the symmetry group $\mathcal{S}'$ and for any $C_i' \in \mathcal{C}_i$ we have $C_i C_i' \in \mathcal{S}'$ by construction, it holds that $\mathcal{S} \subset \overline{\mathcal{G}}$ and $\mathcal{C}_i \subset C_i \overline{\mathcal{G}} \coloneqq \{C_i P | P \in \overline{\mathcal{G}}\}$. This means we may instead sum over the completion of $\mathcal{G}$, noting that any summand not appearing in $\mathcal{T}_{\text{nc}}$ will have zero coefficient:

$$H_{\mathcal{T}_{\text{nc}}} = \sum_{P \in \overline{\mathcal{G}}} \left(h'_P + \sum_{i=1}^{M} h_{P,i} C_i \right) P, \tag{7}$$

where

$$h'_P = \begin{cases} h_P, & P \in \mathcal{S} \\ 0, & \text{otherwise} \end{cases}, \quad h_{P,i} = \begin{cases} h_{C_i P}, & C_i P \in \mathcal{C}_i \\ 0, & \text{otherwise} \end{cases}. \tag{8}$$

We now wish to use this reformulation of the noncontextual Hamiltonian to evaluate expectation values in terms of the parameters $\boldsymbol{\nu} \in \{\pm 1\}^{\times |\mathcal{G}|}$, denoting eigenvalue assignments to the generators $\mathcal{G}$ (sectors), and $\boldsymbol{r} \in \mathbb{R}^M$, a unit vector weighting the class representatives.

We define expectation values with respect to a joint probability distribution

$$P(\boldsymbol{\nu}', \boldsymbol{r}' | \boldsymbol{\nu}, \boldsymbol{r}) \coloneqq \frac{\delta_{\boldsymbol{\nu},\boldsymbol{\nu}'}}{2^M} \prod_{i=1}^{M} |r_i + r'_i|, \tag{9}$$

which is a special-case of the phase-space distribution given by Spekkens[4]. Kirby & Love[5] proved the generators take expectation values

$$\langle G \rangle_{(\boldsymbol{\nu},\boldsymbol{r})} = \nu_{f(G)} \;\forall\, G \in \mathcal{G}, \tag{10}$$

recalling that $f : \mathcal{G} \to \mathcal{I}_{\text{stab}}$ is a mapping onto distinct qubit indices, and class representatives

$$\langle C_i \rangle_{(\boldsymbol{\nu},\boldsymbol{r})} = r_i \;\forall\, i \in \{1, \ldots, M\}. \tag{11}$$

Observe that

$$\langle C(\boldsymbol{r}) \rangle_{(\boldsymbol{\nu},\boldsymbol{r})} = \sum_{i=1}^{M} r_i^2 = |\boldsymbol{r}| = +1, \tag{12}$$

implying any quantum state consistent with the noncontextual state $(\boldsymbol{\nu}, \boldsymbol{r})$ must be stabilized by $C(\boldsymbol{r})$.

Putting everything together, we can express the expectation value of our noncontextual Hamiltonian as

$$\begin{aligned} \langle H_{\mathcal{T}_{\text{nc}}} \rangle_{(\boldsymbol{\nu},\boldsymbol{r})} &= \sum_{P \in \overline{\mathcal{G}}} \left(h'_P + \sum_{i=1}^{M} h_{P,i} \langle C_i \rangle_{(\boldsymbol{\nu},\boldsymbol{r})} \right) \langle P \rangle_{(\boldsymbol{\nu},\boldsymbol{r})} \\ &= \sum_{P \in \overline{\mathcal{G}}} \left(h'_P + \sum_{i=1}^{M} h_{P,i} r_i \right) \prod_{G \in \mathcal{G}_P} \nu_{f(G)}, \end{aligned} \tag{13}$$

where $\mathcal{G}_P \subset \mathcal{G}$ satisfies $P = \prod_{G \in \mathcal{G}_P} G$. Note also that we have used the fact $\langle AB \rangle = \langle A \rangle \langle B \rangle$ when $[A, B] = 0$.

Taken as a classical optimization problem, minimizing the objective function

$$\eta(\boldsymbol{\nu}, \boldsymbol{r}) \coloneqq \langle H_{\mathcal{T}_{\text{nc}}} \rangle_{(\boldsymbol{\nu},\boldsymbol{r})} \tag{14}$$

is NP-complete in general. Despite this, we expect typical instances to be heuristically solvable by classical methods[5]. If one fixes the ± 1 eigenvalue assignments $\boldsymbol{\nu}$ – a case of identifying the correct symmetry sector – this becomes a convex optimization problem over points of the unit $(M-1)$-sphere.

3 ADAPT-VQE

Adaptive Derivative-Assembled Pseudo-Trotter (ADAPT) VQE[6] is a contemporary method of ansatz construction that retains some problem specificity while resulting in dramatically reduced circuit depths compared to conventional chemically-motivated approaches such as Unitary Coupled Cluster[7,8]. The implementation we describe here is of the qubit-ADAPT-VQE[9] variant, which is centred around an operator pool $\mathcal{O} \subset \mathcal{P}_N$; this could for example consist of excitation terms obtained from a Jordan-Wigner encoded coupled-cluster calculation. Terms are selected from the operator pool to append to a dynamically expanding ansatz in line with a gradient-based argument which we describe in Section 3.2.

3.1 Calculating Gradients

Take an operator $A(\boldsymbol{\theta}) = \sum_k \theta_k P_k$ for $\theta_k \in \mathbb{R}, P_k \in \mathcal{P}_N$, a reference state $|\psi_{\text{ref}}\rangle \in (\mathbb{C}^2)^{\otimes N}$ and define the ansatz state

$$|\psi(\boldsymbol{\theta})\rangle = e^{iA(\boldsymbol{\theta})} |\psi_{\text{ref}}\rangle . \tag{15}$$

Application of the chain rule yields

$$\frac{\partial}{\partial \theta_k} |\psi(\boldsymbol{\theta})\rangle = iP_k |\psi(\boldsymbol{\theta})\rangle \tag{16}$$

and combining this with the product rule, we have

$$\begin{aligned}\frac{\partial}{\partial\theta_k}\langle H\rangle_{\psi(\boldsymbol{\theta})} &= \langle\psi(\boldsymbol{\theta})|\left[-iP_kH + iHP_k\right]|\psi(\boldsymbol{\theta})\rangle \\ &= i\left\langle[H,P_k]\right\rangle_{\psi(\boldsymbol{\theta})};\end{aligned}\tag{17}$$

this is the formulation proposed by Grimsley et al.[6]. However, if one observes that

$$\begin{aligned}i[H,P_k] &= \frac{1}{2}\big[(I-iP_k)H(I+iP_k)-(I+iP_k)H(I-iP_k)\big] \\ &= e^{-i\frac{\pi}{4}P_k}He^{i\frac{\pi}{4}P_k} - e^{i\frac{\pi}{4}P_k}He^{-i\frac{\pi}{4}P_k}\end{aligned}\tag{18}$$

then we recover the parameter shift rule of Parrish et al.[10], namely

$$\frac{\partial}{\partial\theta_k}\langle H\rangle_{\psi(\boldsymbol{\theta})} = \langle H\rangle_{e^{i\frac{\pi}{4}P_k}\psi(\boldsymbol{\theta})} - \langle H\rangle_{e^{-i\frac{\pi}{4}P_k}\psi(\boldsymbol{\theta})}.\tag{19}$$

Therefore, we may evaluate the expectation value $\langle H\rangle_{\psi(\boldsymbol{\theta})}$ at $\theta_k+\frac{\pi}{4}$ and $\theta_k-\frac{\pi}{4}$, with the partial gradient with respect to θ_k being their difference. We opt for the latter method of gradient calculation; this allows us to avoid storing a large collection of commutators in memory – each requiring decomposition into qubit-wise commuting measurement groups – at the expense of one additional expectation value calculation per partial gradient.

3.2 The Algorithm

We shall define the algorithm iteratively, initializing a reference state $|\psi_0\rangle := |\psi_{\text{ref}}\rangle$ and describing mathematically how one obtains $|\psi_{k+1}\rangle$ from the k-th ADAPT-VQE cycle. Let us define the function

$$g_P^{(k)}(\theta) := \langle\psi_k|\,e^{-i\theta P}He^{i\theta P}\,|\psi_k\rangle\tag{20}$$

and

$$\begin{aligned}G_P^{(k)}(\theta) &:= \frac{\partial}{\partial\theta}\langle H\rangle_{e^{i\theta P}\psi_k} \\ &= g_P^{(k)}\Big(\theta+\frac{\pi}{4}\Big) - g_P^{(k)}\Big(\theta-\frac{\pi}{4}\Big)\end{aligned}\tag{21}$$

by (19).

Then, identifying the pool operator that yields the largest gradient in magnitude at $\theta = 0$,

$$P_{k+1} = \underset{P \in \mathcal{O}}{\arg\max} \left| G_P^{(k)}(0) \right|, \tag{22}$$

we construct the expanded ansatz

$$|\psi_{k+1}(\boldsymbol{\theta}^{(k+1)})\rangle = e^{i\theta_{k+1}P_{k+1}} |\psi_k(\boldsymbol{\theta}^{(k)})\rangle \, . \tag{23}$$

We now perform a VQE simulation to find

$$\boldsymbol{\theta}^{(k+1)}_{\min} = \underset{\boldsymbol{\theta}^{(k+1)} \in \mathbb{R}^{k+1}}{\arg\min} \, \langle H \rangle_{\psi_{k+1}(\boldsymbol{\theta}^{(k+1)})} \tag{24}$$

and finally set

$$|\psi_{k+1}\rangle = |\psi_{k+1}(\boldsymbol{\theta}^{(k+1)}_{\min})\rangle \, . \tag{25}$$

This process is iterated until some termination condition is satisfied, for example when the largest gradient $\max_{P \in \mathcal{O}} \left| G_P^{(k)}(0) \right|$ falls below a predefined threshold.

We note a few properties of ADAPT-VQE. First of all, in theory, the minimized energy $\langle H \rangle_{\psi_k(\boldsymbol{\theta}^{(k)}_{\min})}$ will decrease monotonically with increasing $k \in \mathbb{N}$. In practice, the ansatz complexity also increases and therefore optimization becomes increasingly demanding, meaning it is possible the true minimum is not found. In such cases, it is possible for the $(k+1)$-th iterate to yield higher energy than the k-th.

Secondly, the operator pool $\mathcal{O}$ is never exhausted, meaning it is possible to select the same term more than once. This gives rise to the 'Pseudo-Trotter' description of ADAPT-VQE, in the sense that it somewhat resembles the Trotter expansion (2) but with only select terms duplicated.

References

(1) Nielsen, M. A.; Chuang, I. L. *Quantum Computation and Quantum Information: 10th Anniversary Edition*; Cambridge University Press, 2010.

(2) Tranter, A.; Love, P. J.; Mintert, F.; Wiebe, N.; Coveney, P. V. Ordering of Trotterization: Impact on errors in quantum simulation of electronic structure. *Entropy* **2019**, *21*, 1218.

(3) Grimsley, H. R.; Claudino, D.; Economou, S. E.; Barnes, E.; Mayhall, N. J. Is the Trotterized UCCSD Ansatz chemically well-defined? *Journal of Chemical Theory and Computation* **2019**, *16*, 1–6.

(4) Spekkens, R. W. *Quantum Theory: Informational Foundations and Foils*; Springer Netherlands: Dordrecht, 2016; pp 83–135.

(5) Kirby, W. M.; Love, P. J. Classical simulation of noncontextual Pauli Hamiltonians. *Physical Review A* **2020**, *102*, 032418.

(6) Grimsley, H. R.; Economou, S. E.; Barnes, E.; Mayhall, N. J. An adaptive variational algorithm for exact molecular simulations on a quantum computer. *Nature Communications* **2019**, *10*, 1–9.

(7) Taube, A. G.; Bartlett, R. J. New perspectives on unitary coupled-cluster theory. *International Journal of Quantum Chemistry* **2006**, *106*, 3393–3401.

(8) Romero, J.; Babbush, R.; McClean, J. R.; Hempel, C.; Love, P. J.; Aspuru-Guzik, A. Strategies for quantum computing molecular energies using the unitary coupled cluster ansatz. *Quantum Science and Technology* **2018**, *4*, 014008.

(9) Tang, H. L.; Shkolnikov, V.; Barron, G. S.; Grimsley, H. R.; Mayhall, N. J.; Barnes, E.; Economou, S. E. qubit-adapt-vqe: An adaptive algorithm for constructing hardware-efficient ansätze on a quantum processor. *PRX Quantum* **2021**, *2*, 020310.

(10) Parrish, R. M.; Hohenstein, E. G.; McMahon, P. L.; Martinez, T. J. Hybrid quantum/classical derivative theory: Analytical gradients and excited-state dynamics for the multistate contracted variational quantum eigensolver. *arXiv preprint* **2019**,
